# Case study: organizing outpatient pharmacological treatment of bipolar disorder in autism, intellectual disability and Phelan-McDermid syndrome (22q13.3 deletion syndrome)

**DOI:** 10.1080/20473869.2020.1756113

**Published:** 2020-06-09

**Authors:** Anne Langseth Rysstad, Arvid Nikolai Kildahl, Jon Olav Skavhaug, Monica Stolen Dønnum, Sissel Berge Helverschou

**Affiliations:** 1 Section for Intellectual Disabilities and Autism, Vestre Viken Hospital Trust, Asker, Norway; 2 Regional Section Mental Health, Intellectual Disabilities/Autism, Oslo University Hospital, Oslo, Norway; 3 NevSom Norwegian Centre of Expertise for Neurodevelopmental Disorders and Hypersomnias, Oslo University Hospital, Oslo, Norway

**Keywords:** phelan-McDermid syndrome, 22q13.3 deletion syndrome, autism spectrum disorder, intellectual disability, bipolar disorder, treatment

## Abstract

Phelan-McDermid syndrome (PHMDS)/22q13.3 deletion syndrome is a rare genetic disorder associated with autism spectrum disorder (ASD), intellectual disability (ID), and bipolar disorder. While numerous cases have been reported describing successful pharmacological treatment of bipolar disorder in PHMDS, there is currently little guidance available on how to organize and execute such treatment. The aim of the current case study was to explore how pharmacological treatment of bipolar disorder in PHMDS may be organized and evaluated in an outpatient setting. Through a complex process of try and fail, including systematic evaluation of any change to the intervention and never implementing more than one change at the time, the patient gradually improved, regaining his communicative and adaptive skills. Four years passed from referral to this result was achieved. Organizing assessment and treatment as a collaborative effort involving specialized mental health professionals, professional caregivers and the patient’s family proved feasible. Many of the challenges present in assessment of psychiatric disorder in individuals with ASD and ID are likely to be present also in evaluation of treatment effects, particularly in disorders where symptoms occur in phases. The approach described in the current paper may contribute to reducing the impact of these challenges.

## Introduction

While individuals with autism spectrum disorder (ASD) and intellectual disability (ID) are susceptible to the same set of psychiatric disorders as the population in general (Rosen *et al*. 2018, Bakken *et al*. 2016a), identification and treatment of these disorders in this population is challenging. Several factors contribute to these challenges: ASD and ID may both influence the behavioural manifestation of psychiatric symptoms (Rosen *et al*. 2018, Bakken *et al*. 2016a, Helverschou *et al*. 2011), and individuals with ASD and ID often have limited verbal skills and difficulties conveying their own experience of symptoms (Bakken *et al*. 2016a, Helverschou *et al*. 2011). Few instruments are available for use in assessments (Rosen *et al*. 2018, Bakken *et al*. 2016a), and there is a risk of diagnostic overshadowing (Reiss *et al*. 1982), where symptoms of psychiatric disorder is misinterpreted and attributed, instead, to ASD and/or ID (Helverschou *et al*. 2011). Moreover, ASD and ID both seem to be associated with increased risk of developing psychiatric disorder (Rosen *et al*. 2018, Bakken *et al*. 2016a). This includes specific genetic syndromes associated with ASD and ID making individuals particularly vulnerable to specific psychiatric disorders (Kolevzon *et al*. 2019, Crawford et al., 2017, Monks *et al*. 2014).

Phelan-McDermid syndrome (PHMDS) or 22q13.3 deletion syndrome (OMIM **606232**) is a rare, genetic disorder caused by a haploinsufficiency of the *SHANK3* gene resulting from either a point mutation or an heterozygous deletion on the distal long arm of chromosome 22 (Kolevzon *et al*. 2019, Soorya *et al*. 2013, Phelan *et al.* 2011). PHMDS is typically characterized by co-occurring ASD and moderate to severe ID (Droogmans *et al*. 2019, Richards *et al*. 2017, Sarasua *et al*. 2014, Soorya *et al*. 2013), as well as hypotonia and speech impairment (Kolevzon *et al*. 2014). However, recent studies have described considerable phenotypic variation in individuals with the deletion, including individuals who do not have ID (Samogy-Costa *et al.* 2019, Kolevzon *et al*. 2019).

Several studies have reported of apparent developmental regressions, i.e. loss of communicative and adaptive skills, occurring in adulthood in individuals with PHMDS (Kohlenberg *et al*. 2020, Droogmans *et al*. 2019,Verhoeven *et al*. 2020, Jungová *et al*. 2018, Rowland *et al*. 2018, De Rubeis *et al*. 2018, Tabet *et al.* 2017, Egger *et al*. 2017, Breckpot *et al.* 2016, Egger *et al*. 2016, Messias *et al*. 2013, Verhoeven *et al*. 2012a, 2012b, Denayer *et al*. 2012, Bonaglia *et al.* 2011, Nawab *et al*. 2007, Anderlid *et al*. 2002). Of the 56 published PHMDS cases involving apparent loss of skills in adolescence or adulthood identified by Kolevzon *et al*. (2019), 30 (54%) were deemed most likely to meet criteria for bipolar disorder, and recent findings indicate that bipolar disorder may be one of the more commonly co-occurring medical conditions in PHMDS (Kohlenberg *et al*. 2020, Verhoeven *et al*. 2020, Kolevzon *et al*. 2019). Even though recognition and identification of bipolar disorder in individuals with PHMDS may be challenging (Kohlenberg *et al*. 2020, Kolevzon *et al*. 2019, Kildahl *et al*. 2020), assessment methodologies used for individuals with ASD and ID in general seem to be applicable also for individuals with PHMDS (Kildahl *et al*. 2020, Bakken *et al*. 2016a, Helverschou *et al*. 2011). However, as assessment methodologies in the published reports have varied considerably (Kolevzon *et al*. 2019), it is currently challenging to draw general conclusions regarding the presentation of bipolar disorder in PHMDS.

Current knowledge regarding organization of pharmacological treatment is sparse for psychiatric disorder in ASD and ID in general (Trollor et al., 2016), and for PHMDS in particular (Kohlenberg *et al*. 2020, Kolevzon *et al*. 2019). Moreover, resources for treatment of bipolar disorder in individuals with ID (Tromans *et al*. 2020) or ASD (Vannucchi et al. 2019) provide guidelines for drug choice and monitoring, but leave little guidance for clinicians, caregivers and families on how to organize and systematically evaluate such treatments in outpatient settings. For PHMDS specifically, descriptions involving successful psychopharmacological treatments of regression, bipolar disorder, and other psychiatric conditions (Verhoeven *et al*. 2020, Rowland *et al*. 2018, Egger *et al*. 2017, 2016, Serret *et al*. 2015, Denayer *et al*. 2012, Verhoeven *et al*. 2012a, 2012b, Vucurovic *et al*. 2012) are frequently limited to a primary focus on drug choice and dosage. Moreover, these reports have rarely included use of systematic assessment tools in the evaluation of treatment, making treatment effects challenging to compare across studies. Furthermore, the organization, contextual factors, and time involved in these treatments have been scarcely described, again leaving little guidance for clinicians, caregivers and families on how to organize and evaluate treatment in outpatient settings, as well as how quickly to expect treatment to be effective.

The aim of the current study was to explore and describe how pharmacological treatment of bipolar disorder in PHMDS may be organized and evaluated in an outpatient clinic, including exploration of the entire course of treatment, the time involved, and practices aiding in the collaboration between family, specialist mental health services, and professional caregivers.

## Materials and methods

### Design

To be able to explore the organization of treatment over time, case study methodology was chosen (Yin 2014). There is a distinct lack of reports on real-world investigation of ASD intervention, and case studies are seen as presenting important contributions to this field (Bulkeley *et al*. 2013). This also applies to treatment of psychiatric disorder in this population, particularly for individuals with co-occurring ID.

### Measures

In line with recent recommendations for psychiatric assessment in ASD and ID, a combination of ASD-specific and conventional assessment tools were used (Helverschou et al., in press). The initial assessment included use of several conventional assessment tools: the Mini Neuropsychiatric Interview (MINI) (Sheehan *et al*. 1998), the Montgomery Åsberg Depression Rating Scale (MADRS) (Montgomery et al. 1979), the Young Mania Rating Scale (YMRS) (Young *et al*. 1978), and the PANSS-8 (Lin *et al*. 2018, Andreasen *et al*. 2005), an abbreviated version of the Positive and Negative Syndrome Scale (PANSS) (Kay *et al*. 1987). Except for the MADRS, all of these instruments have previously been described as useful in a previous single-case study describing psychiatric assessment in PHMDS (Kildahl *et al*. 2020). Conventional assessment tools were all completed as interviews with the patient’s professional caregivers in the manner described by Kildahl *et al*. (2017). However, because these assessment tools have all been developed for use with self-report, their use with informants may have compromised their validity and results have to be interpreted with caution.

The Psychopathology in Autism Checklist (PAC) (Helverschou *et al*. 2009) is a 42-item checklist developed for psychiatric screening in individuals with ASD and ID, showing good reliability in clinical populations. The PAC was developed by identifying items describing symptoms of psychiatric disorder which do not overlap with symptoms of ASD, in order to overcome some of the challenges inherent to these assessments (Helverschou *et al*. 2009). In the current case, the PAC was completed by the patient’s caregivers and his family, and this was repeated several times during treatment. PAC scores from throughout the treatment are presented in Table 2, while scores from the MADRS and the PANSS reflecting depressive and psychotic symptoms are presented in Table 3.

**Table 2.** PAC Scores Throughout Treatment.

	Initial assessment (November, year 1)		Manic episode (July, year 3)	Follow-up (August, year 5)	
	Family	Caregivers	Caregivers	Family	Caregivers
General difficulties	2.8*	2.2*	2.2*	1.2	1.5
Psychosis	3.3*	2.4*	3.4*	1.0	1.5
Obsessive-compulsive disorder	1.9	1.7	1.3	1.3	1.3
Depression	3.6*	2.7*	2.7*	1.1	1.7
Anxiety	1.8*	1.8*	3.8*	1.0	1.2

Note. The Psychopathology in Autism Checklist (PAC) was scored by the patient’s mother and sister in cooperation, and separately by two professional caregivers. For the latter, the average for each score is given. Scores on or above cut-offs are indicated by an asterisk (*). Cut-offs are derived from studies involving individuals with co-occurring ASD and ID (Helverschou et al. 2009, see also Bakken et al. 2010).

**Table 3.** PANSS-8 and MADRS Scores Throughout Treatment.

	Initial assessment (Nov. year 1)	After 1 year (Dec. year 2)	After 1,5 year (Feb. year 3)	Follow-up (Aug. year 5)
**PANSS-8**				
Total score	49	28	22	12
P1 Delusions	6*	3	3	2
P2 Conceptual disorganization	7*	4*	3	1
P3 Hallucinatory behaviour	3	2	2	1
N1 Blunted affect	7*	3	3	2
N4 Social withdrawal	7*	4*	3	2
N6 Lack of spontaneity and flow of conversation	7*	4*	3	1
G5 Mannerisms and posturing	7*	4*	2	1
G9 Unusual thought content	5*	4*	3	2
**MADRS**				
Total score	49	17	15	5

Note. PANSS-8 is an abbreviated 8-item version of the Positive and Negative Syndrome Scale (Andreasen et al. 2005, Kay et al. 1987). Scores for the individual items and the total score is given. In typically developing individuals, item scores of 4 or more usually indicate significant problems and are marked by an asterisk (*). Though norms have not been adapted for individuals with ASD and ID and scores need to be interpreted with caution, these instruments were both completed by mental health professionals with extensive experience in ASD and ID, taking account of the patient’s underlying conditions. MADRS is the Montgomery-Åsberg Depression Rating Scale (Montgomery *et al.*, 1979). In typically developing individuals, a score above 35 indicates a severe depressive disorder, while scores between 7 and 19 indicates mild depressive symptomatology.

### Research ethics

The study was approved by the Data Protection Officer at the Vestre Viken Hospital Trust. The patient was not able to give consent, but consent was provided by his mother/legal guardian. The patient has been anonymized, and his family has read and approved the manuscript prior to submission.

## Case description

At the time of the referral “Jonathan” was a young man in his late twenties. He had previous diagnoses of ASD and moderate ID. PHMDS was diagnosed during the present assessment using genome-wide array comparative genomic hybridization (aCGH) (Sureprint G3 Human CGH microarray, 180 k, from Agilent Technologies, CA, USA), showing an approximately 50** **kb deletion on chromosome 22 (22q13.33). MLPA (P188, MCR-Holland) confirmed the deletion, with a proximal breakpoint between intron 3 and 9 in *SHANK3*, extending to and including *ACR* and *RABL2B*.

Jonathan showed developmental delay from an early age, walking and speaking late. He received a diagnosis of ID in preschool age, which was specified at moderate ID in his early teens. ASD was also diagnosed in his early teens. Jonathan developed complex verbal utterances around the age of six, but his verbal communication was characterised by a lack of perspective taking and a tendency to talk only about the things that interested him. His verbal language included frequent use of idiosyncrasies and professional care staff would often struggle to understand him until they had worked with him for some time. Jonathan had some adaptive skills, but needed help with most things in daily life, including personal hygiene and preparing meals. Jonathan had repetitive motor behaviours typically seen in ASD in childhood, including hand flapping. However, possibly atypical for PHMDS (Richards *et al*. 2017), these behaviours disappeared by Jonathan’s teenage years. His stereotypic behaviours in adulthood were described more in terms of insistence on sameness, specifically an obsession that everything should be in its rightful place at all times.

Jonathan grew up living with his parents and two older sisters in an affluent neighbourhood. He had received special education, usually individually or in small groups, throughout his school years. There had been periods of challenging behaviour and restlessness throughout Jonathan’s life, including hitting and pushing people, as well as milder forms of self-injurious behaviour, such as skin picking. Jonathan moved into an insufficiently adapted group home at 18, leading to increases in challenging behaviour. This improved again when he moved to a better suited group home at age 20.

A few years prior to the current referral, Jonathan had undergone a mental health assessment concluding that he had a major depressive episode. During this episode, he displayed more negative mood and greater difficulties with emotion regulation, becoming more easily frustrated and irritable. He also had sleep difficulties, spoke less than usual and showed less initiative. During this episode, Jonathan was prescribed risperidone (2** **mg), which had to be reduced to a lower dosage (1** **mg) due to Jonathan developing bradykinesia as a side effect. Jonathan also had digestive problems, including frequent obstipation, as well as pollen allergies.

### Referral and assessment

Leading up to the current referral, Jonathan’s family and his professional caregivers for months grew increasingly concerned about his behaviour. In June, Jonathan showed less initiative and seemed more negative and less happy. He started talking about topics that were unusual for him, particularly fires and death, and seemed more stressed and agitated. These difficulties gradually worsened, and Jonathan started displaying difficulties completing his regular tasks. For instance, when clearing the table, he would seem agitated and disorganized. Several times, he threw cutlery and tableware in the garbage instead of the dishwasher and put his used napkin in the fridge. These were tasks Jonathan usually mastered, and he would be quite peculiar about them, often correcting staff if they put something in the ‘wrong’ place in his dishwasher.

In July, Jonathan had an episode where he slept very little over several days and completely stopped communicating verbally. This was highly unusual for him. He started speaking again when sleep was re-established after a few days, but his preoccupations with fires and death continued, as did his agitation, lack of initiative, and negative mood. When family visited, he often seemed positively surprised, occasionally exclaiming: ‘But mom, you weren’t dead!’ During these months, Jonathan experienced a weight loss of around 20** **kg.

Following these concerns, Jonathan was referred by his primary physician to a specialized mental health outpatient clinic for individuals with ASD and/or ID. Following a thorough assessment involving the instruments described above, it was concluded that Jonathan met criteria for a bipolar disorder. Symptoms prior to referral were understood as a major depressive episode with psychotic symptoms, and ideas that his family had died were understood as depressive delusions. It has previously been described (Kildahl *et al*. 2017) that the manifestation of repetitive behaviours may change with psychiatric symptom load. Jonathan did not develop any repetitive motor behaviours during this period, but his insistence on things being in their rightful place seemed to intensify as symptoms developed - up to a certain point where his behaviour became disorganized and he no longer managed to keep track of where things belonged. Thus, at first Jonathan would no longer allow guests to finish their meal before he cleared the table, occasionally putting the dirty tableware directly back into the cupboard. When symptoms worsened, he sometimes seemed passive and not to care, while he at other times would try to put tableware in the rubbish bin.

### Organization of treatment

The treatment plan was made in collaboration between Jonathan’s family, the professional caregivers in his home, and the specialized outpatient clinic. Assessment and treatment was organized as a series of meetings between Jonathan’s mother and sister, the two professional caregivers who had the main responsibility for his care, and a psychiatrist and an intellectual disability nurse from the specialized outpatient clinic. Initially, these meetings took place every three months. Later, when Jonathan’s condition had become more stable (from *year 4* in the presentation below), they were organized twice yearly. Meetings included discussion of the status quo, evaluation of any change implemented since the last meeting, and agreeing on changes in the time leading up to the next meeting. The intellectual disability nurse from the outpatient clinic visited Jonathan several times between meetings, advising his care staff. There was also frequent contact between Jonathan’s family and his caregivers.

Even if cooperation was emphasized by all involved parties, there was a clear distribution of roles. The psychiatrist had the final say in any decision regarding pharmacological treatment, and contributed with medical and pharmaceutical knowledge, while the professional caregivers had overview of Jonathan’s current status and the day-to-day monitoring of behaviour. Jonathan’s family contributed with their interpretation and understanding of Jonathan’s behaviour, having known him his whole life. A combination of all these approaches were often necessary throughout the treatment to separate behaviours associated with bipolar disorder and treatment intervention from those associated with Jonathan’s ASD, ID, personality, and general demeanour.

Based on information from Jonathan’s family, it was assumed that he would be sensitive to changes to internal states and bodily sensations. Somatic health problems, including back pain, obstipation, and fever were all described as usually being accompanied by changes to Jonathan’s behaviour. It was therefore assumed that changes to Jonathan’s medication might in itself affect Jonathan’s behaviour, complicating evaluation of treatment. Experienced discomfort from side-effects, for instance, was assumed to be associated with a risk of Jonathan becoming restless. Similarly, if Jonathan experienced changes to his thoughts and feelings that he did not understand, it was assumed that these could make him feel insecure. It was therefore concluded that it would be important to make sufficient time from any change to Jonathan’s medication to its evaluation, allowing Jonathan time to adjust. Similarly, it was agreed that changes to medication should preferably be avoided close to events that typically led to changes to Jonathan’s behaviour: Christmas, summer holidays, and the pollen season. The importance of making only one change at the time was also emphasized. Dosage was regularly monitored via blood samples, and Jonathan’s circadian rhythm was monitored daily by caregivers using a form and coloured markers.

During assessment, Jonathan’s professional caregivers and the intellectual disability nurse from the specialist outpatient clinic devised an intervention plan adapted for his different symptom phases. It included descriptions of the various phases aimed to make recognition easy for all caregivers, as well as descriptions of how care should be adapted during these different phases. The plan was also discussed and evaluated in meetings with Jonathan’s family. If Jonathan showed symptoms of restlessness and disorganization and had not slept, for instance, staff would place less demands on Jonathan, trying to help him calm down and providing more assistance than usual for practical tasks. The plan was considered an important tool to ensure that Jonathan’s care was adapted to his varying difficulties in a consistent way across caregivers. A crisis management plan (see Mohiuddin *et al*. 2011) was devised for situations where Jonathan showed symptoms of mania.

No new behavioural therapeutic interventions were introduced during the treatment period. In accordance with principles from mental health nursing (Bakken et al. 2016b), Jonathan’s professional caregivers focused on adapting demands, tasks and activities to Jonathan’s current status and symptom load. This included Jonathan participating in his regular daily activities; going to a day centre, going for walks, car and bike rides, cleaning his yard, watching TV, getting visits from family, and participating in planning meals. Because Jonathan showed a reduced number of initiatives, his caregivers strove to facilitate Jonathan making choices and validating (Bakken *et al*. 2017) any initiative he made. They also continued to make use of alternative and augmentative communication tools that Jonathan had previously made use of, including a pictogram-based day planner (Howlin 2006).

Finally, prior to psychopharmacological intervention, Jonathan’s drug metabolization in the CYP450 system was investigated (Stahl 2014). This showed that Jonathan had a hetereozygote mutation in the CYP2D6. This was deemed as unlikely to affect his metabolization of psychopharmacological treatment, but it was noted that Jonathan previously had developed side effects at a surprisingly low dosage of a drug metabolized by this particular enzyme system (risperidone).

### Treatment

#### For a complete timeline of the treatment, see Table 1.

##### Year 1

Jonathan was referred to the outpatient clinic in September. Due to his previous reaction to risperidone, aripripazole (5** **mg) was initiated in an attempt to alleviate psychotic symptoms shortly after referral.

##### Year 2

In January, aripripazole was moved from mornings to evenings due to suspected sedation during the day. In February, risperidone dosage was reduced from 1** **mg to 0.5** **mg. Allowing time for Easter, aripripazole dosage was increased to 10** **mg in April, and risperidone completely discontinued in May. Because sleep was still an issue, Melatonin was initiated in late May.

The results from the genetic assessment came back in March, leading to the recommended somatic assessments being carried out (Kolevzon *et al*. 2014). The diagnosis of PHMDS further led to a hypothesis that some of Jonathan’s restlessness and discomfort might be associated with gastrointestinal reflux, as vomiting had been a frequent issue during Jonathan’s childhood. This led to the introduction of pantoprazole sodium, which seemed to have a positive effect on Jonathan and seemed to be associated with a slight reduction in utterances about fires and burning.

In June, aripripazole dosage had to be reduced to 5** **mg due to Jonathan developing tremor as a side effect. These tremors subsided shortly after the reduction of dosage. Though it was agreed that antipsychotic medication had a positive effect on Jonathan’s behaviour, this effect was deemed to be minor by his family and professional caregivers, with Jonathan still having a considerable symptom load (see Tables 2 and 3). In July, Jonathan developed symptoms of severe restlessness, continually walking around his apartment, significant reduction of verbal language, and very little or no sleep for several days. Jonathan seemed driven, stressed, uncomfortable and frightened during this episode, which was assessed by the psychiatrist to constitute a manic episode. Following this episode, the final diagnosis of bipolar disorder was made in August.

Following a review of the literature on treatment of bipolar disorder in PHMDS (e.g. Egger *et al*. 2017, 2016, Verhoeven *et al*. 2012b), it was agreed to introduce a mood stabilizing agent along with the antipsychotic. Lamotrigine was chosen due to the severity of depressive symptoms at referral and the apparent persistence of these symptoms (Calabrese *et al.* 2003). It was introduced in a dosage of 50** **mg and then gradually increased during the following autumn until a dosage of 150** **mg x2 was reached.

##### Year 3

Jonathan’s sleeping patterns seemed to be unaffected by the introduction of melatonin, and it was therefore discontinued in March. However, shortly after this, Jonathan developed more symptoms of restlessness and difficulties sleeping and apparent symptoms of hypomania – i.e. not as severe as previous manic episodes. Thus, in April Melatonin was re-instated and risperidone 1** **mg was given as optional medication in instances of severe restlessness. The effect of this, however, was unclear. This use of risperidone was therefore discontinued in May. The dosage of Lamotrigine was increased in April, reaching 200mg given twice daily.

In June, Jonathan again developed symptoms of mania. He had several days with no sleep, continually wandering around his apartment, communicating little and seeming driven and intense. He showed severely disorganized behaviour, and had difficulties cooperating in basic tasks such as getting dressed, eating, going to the toilet and having a bath. Symptoms equalled an YMRS score of 44, which in typically developing individuals would indicate severe mania (Young *et al*. 1978). Staffing was doubled, for both Jonathan’s and staffs’ safety. Olanzapine was introduced in a dosage of 5** **mg. While Jonathan’s symptoms were unaffected by this, he did not show any initial side-effects and the dosage was increased twice. At a total dosage of 15** **mg olanzapine for two consecutive days, with the addition of 5** **mg nitrazepam for sleep, Jonathan slept for more than 12** **h and mania symptoms gradually subsided over a few days. Nitrazepam was discontinued after three full nights’ sleep.

In August Jonathan ate well, slept more or less as usual and again communicated more verbally. Unusually for him, however, he still seemed anxious when left alone. Olanzapine dosage was reduced to 5** **mg + 5** **mg. Due to the severity of the manic episodes and the risk of new episodes, it was agreed to keep Jonathan’s pharmacological treatment, sleeping pattern and general surroundings as stable as possible during the following autumn and winter. Zopiklone was introduced in an attempt to further stabilise Jonathan’s sleeping patterns, but seemed to have little effect and was changed to alimemazine 10** **mg, then 20** **mg. Melatonin was discontinued. In a further attempt to ensure stable sleeping patterns, the entire olanzapine dosage was moved to the evening. At this point, Jonathan thus received 10** **mg. olanzapine in the evenings, lamotrigine 200** **mg x2, alimemazine 20** **mg, and aripripazole 5** **mg, as well as pantoprazole sodium for gastrointestinal reflux, and cetirizine for his allergies.

##### Year 4

It was unclear whether the remaining dosage of aripripazole was helpful to Jonathan, but due to the apparently accentuated risk of developing mania during summer it was agreed not to make any changes to his medication during the spring. The crisis management plan was updated, but Jonathan did not develop mania symptoms in the summer; he slept well and did not show increased restlessness. Aripripazole dosage was reduced from 5** **mg to 2.5** **mg in September and discontinued in December. It was also suspected that the use of alimemazine gave Jonathan slight hang-overs and might negatively affect his cognitive capacity. Alimemazine was therefore gradually discontinued without any apparent negative changes to Jonathan’s sleeping patterns. At the end of the year, Jonathan’s medication comprised olanzapine 10** **mg, lamotrigine 200** **mg x2, pantoprazole sodium and cetirizine.

##### Year 5

In case aripripazole had been working as a protective factor for mania, it was agreed not to make any further changes to Jonathan’s medication during spring. The crisis management plan was updated, but did not come into effect, as Jonathan did not develop symptoms of mania. In the autumn, olanzapine was further reduced to 7.5** **mg, resulting in Jonathan seeming slightly more socially present and aware of his surroundings, more frequently commenting on things he finds interesting, and making more conversational initiatives. However, a full evaluation of this change will not be possible until the summer of the next year.

## Results

Four years after referral, Jonathan has regained most of his previous functioning and he has had two consecutive summers without symptoms of severe psychopathology. The return of adaptive and communicative functioning has been gradual, with no leaps associated with changes to his medication. In general, these changes seem to have followed a pattern where change of medication first seemed to affect symptom load, and improvement of adaptive and communicative skills followed gradually. The current psychopharmacological treatment, with concurrent use of one mood stabilizer and an atypical antipsychotic, is in line with previous descriptions of pharmacological treatment of bipolar disorder in PHMDS (Verhoeven *et al*. 2020, Kolevzon *et al*. 2019). However, arriving at this combination of drugs required a complex process of try and fail, which in turn required close cooperation between family, professional caregivers, and the specialized outpatient clinic. The clinical impression of improvement is in line with current scores of the PAC (Table 2). Though he still has challenges related to regulation of emotion and behaviour, Jonathan’s mother recently expressed that ‘we have gotten him back now’. As for his insistence that things should be in their rightful place, Jonathan is now back to accepting that guests may finish their meals before he takes away their dishes and puts them in the dishwasher.

## Discussion

Identification and treatment of bipolar disorder in PHMDS required a systematic, collaborative effort involving the patient’s family and his professional caregivers, as well as specialized mental health professionals. The organization of treatment as a series of meetings where pharmacological and other interventions were planned and evaluated worked well, easing communication between the involved parties. Noticeably, four years passed from referral until the involved parties considered treatment to have been successful.

While the pharmacological treatment administered is typical for bipolar disorder, the manner in which it was organized and evaluated was not. Drugs were initiated at a lower dosage than usual, and dosages were increased more slowly. Even if individuals with PHMDS and others with ASD and ID are often unable to express how they subjectively experience changes to pharmacological treatment, they are likely to be affected by side effects or other experiences of discomfort similarly to others. Furthermore, even for positive effects, individuals with co-occurring ASD and ID may not be able to understand what is happening to them and positive treatment effects may thus be experienced as strange or frightening for these individuals. This suggests that it may be important to allow for enough time to pass before evaluating the effect of medication change, to avoid actual effects and side effects being confused with behavioural change associated with the subjective experience of the change itself.

Effects of treatment in the current case were rarely immediately observable. Rather, these effects seemed to follow a pattern where there was an initial, gradual effect on symptom load, which was followed by gradual improvements to adaptive and communicative functioning. Improvements in communicative and adaptive functioning in the current patient have been gradual for *two years* following his last manic episode. This further underlines the importance of making enough time before evaluating pharmacological intervention in mental health problems in PHMDS. The exception to this pattern was during episodes of mania, where effects of increased antipsychotics and sleep medication were more or less instant when reaching a certain dosage. Furthermore, the use of an atypical antipsychotic as prophylaxis to prevent development of new episodes of mania could not be adequately evaluated until the following summer had passed, as it was during summers the patient seemed to be at the highest risk. In the evaluation of changes to pharmacological treatment, it was also necessary to take account of contextual factors that might have influenced the patient’s behaviour. Together, this underlines that treatment of bipolar disorder in PHMDS is a time-consuming process, as well as the importance of using a systematic approach to symptom monitoring and evaluation of treatment effects.

Recent findings indicate that bipolar disorder may be one of the most commonly co-occurring conditions in individuals with PHMDS (Kolevzon *et al*. 2019, Verhoeven *et al*. 2020), indicating a need for further research on the genetic aspects of bipolar disorder in PHMDS. Little is known regarding whether there are specific genetic variants in PHMDS that carry a particularly increased risk of bipolar disorder or loss of skills. As pointed out by Mitz (2019), cases of bipolar disorder in PHMDS have primarily been described in individuals with smaller deletions (see Kolevzon *et al*. 2019). This may be because larger deletions are associated with more severe levels of ID and bipolar disorder thus being more challenging to identify, but it is also possible that the risk of bipolar disorder is specifically associated with changes to the *SHANK3* gene. Consistent with previous findings, the patient in the current study had a smaller deletion. While the pharmacological aspects of treatment of bipolar disorder has been described in previous studies (Rowland *et al*. 2018, Egger *et al*. 2017, 2016, Serret *et al*. 2015, Denayer *et al*. 2012, Verhoeven *et al*. 2012a, 2012b, Vucurovic *et al*. 2012), the current study may provide a starting point in the discussion of how such treatment may be most appropriately organized, and may aid mental health professionals in conveying hope to families and professional caregivers that treatment may be successful even though this is not always immediately obvious.

Some studies have described the bipolar disorder occurring in individuals with PHMDS as ‘atypical’ (Verhoeven *et al*. 2020), but neither the current pharmacological treatment nor the ones previously described (Kolevzon *et al*. 2019, Verhoeven *et al*. 2020) seem to differ substantially from what is applied in bipolar disorder in the general population (Connolly and Thase, 2011). Furthermore, symptoms in the current case were measurable using conventional assessment tools. This is in line with findings from at least one previous study (Kildahl *et al*. 2020), but further research is needed regarding assessment and treatment of bipolar disorder in individuals with PHMDS.

Treatment was organized as a collaborative effort, with family, professional caregivers, and specialized mental health professionals participating on an equal footing. Regular meetings provided opportunities to evaluate prior interventions and discuss future ones, as well as the prioritizing the sequence of these. These discussions also ensured that all involved parties contributed with their specific knowledge of the patient, observations and experience of the status quo. The regularity of these meetings was seen both as aiding in communication and reducing the risk of misunderstandings and conflict, by everyone involved meeting face to face and neither family nor professional caregivers having to rely on second-hand information from the specialized mental health professionals. This collaboration is likely to have been particularly important in attempts to make sense of the changes to the patient’s stereotypic behaviour and his insistence on sameness.

In our view, the current organization served as an important means to overcome some of the inherent challenges to evaluating the effects of psychopharmacological treatment in individuals with ASD and ID, and may therefore serve as a template for other mental health professionals facing these challenges. This may be particularly relevant in cases involving psychiatric disorders where symptoms occur in phases, because challenges inherent to both diagnostic assessment and evaluation of treatment in individuals with ASD and ID (Rosen *et al*. 2018, Bakken *et al*. 2016a) are likely to be accentuated for these disorders. When symptoms are naturally varying over time, there may be an *ongoing* risk of misinterpretation involving either diagnostic overshadowing, or wrongly attributing features of ASD or ID to the co-occurring psychiatric disorder. This suggests that measures undertaken to overcome these risks during assessment need to be applied throughout the entire course of treatment in bipolar disorder in ASD and ID. Continually striving to unite the views of family members, professional caregivers, and mental health professionals, may be one approach to reduce these risks.

## Figures and Tables

**Table 1.** Timeline for Symptoms, Measures, and Changes to Pharmacological Treatment.

Year 1	June	Initial worry about changed mood and lack of initiative.
	July	Disorganized behaviour, difficulties completing regular tasks. Several days without verbal communication.
	August	Severe weight loss becoming apparent. Continued disorganized behaviour.
	September	Referral to specialized mental health clinic.
	October	
	November	Aripripazole (5 mg) initiated. PAC, MADRS, PANSS-8 scored.
	December	
Year 2	January	Aripripazole moved from mornings to evenings.
	February	Risperidone reduction (1 mg to 0.5 mg).
	March	Phelan-McDermid syndrome diagnosis obtained. Pantoprazole sodium initiated.
	April	Aripripazole increased (5 mg to 10 mg).
	May	Melatonin initiated. Risperidone discontinued.
	June	Aripripazole dosage decreased (10 mg to 5 mg) due to side effects.
	July	Manic episode.
	August	Bipolar disorder diagnosed.
	September	Lamotrigine initiated (50 mg) and increased (50 mg x2)
	October	Lamotrigine increased (75 mg x2)
	November	Lamotrigine increased twice (100 mg x2, later (125 mg x2)
	December	Lamotrigine increased (150 mg x2). MADRS, PANSS-8 scored.
Year 3	January	
	February	MADRS, PANSS-8 scored.
	March	Melatonin discontinued.
	April	Sleep problems, possible hypomania. Lamotrigine increased (200 mg x2). Melatonin re-instated. Optional risperidone 1 mg initiated.
	May	Optional risperidone discontinued.
	June	Severe manic episode. PAC, YMRS scored. Olanzapine initiated (5 mg, later increased to 10 and finally 15 mg). Nitrazepam (5 mg) initiated, and discontinued after three consecutive nights of sufficient sleep.
	July	Mania symptoms gradually subsiding.
	August	Olanzapine decreased to 5 + 5 mg. Zopiklone initiated, discontinued after six days. Alimemazine initiated (10 mg) and increased (20 mg).
	September	Olanzapine changed from 5 + 5 mg to 10 mg in the evening.
	October	
	November	
	December	
Year 4	January	
	February	
	March	
	April	
	May	
	June	
	July	
	August	
	September	Aripripazole reduced from 5 mg to 2.5 mg.
	October	Alimemazine dosage reduced (10 mg)
	November	Alimemazine discontinued.
	December	Aripripazole discontinued.
Year 5	January	
	February	
	March	
	April	
	May	
	June	
	July	
	August	PAC, MADRS, PANSS-8 scored.
	September	
	October	Olanzapine dosage reduced from 10 mg to 7.5 mg.

Note. PANSS-8 is an abbreviated 8-item version of the Positive and Negative Syndrome Scale (Andreasen et al. 2005, Kay et al. 1987), PAC is the Psychopathology in Autism Checklist (Helverschou et al. 2009), MADRS is the Montgomery-Åsberg Depression Rating Scale (Montgomery and Åsberg 1979), and YMRS is the Young Mania Rating Scale (Young et al. 1978).
